# Rare Detection of Antiviral Functions of Polyclonal IgA Isolated from Plasma and Breast Milk Compartments in Women Chronically Infected with HIV-1

**DOI:** 10.1128/JVI.02084-18

**Published:** 2019-03-21

**Authors:** Matthew Zirui Tay, Erika L. Kunz, Aaron Deal, Lu Zhang, Kelly E. Seaton, Wes Rountree, Joshua A. Eudailey, Jack Heptinstall, Michael D. McRaven, Edgar Matias, Erin McGuire, Nicole L. Yates, Lautaro G. Perez, David C. Montefiori, R. Glenn Overman, Thomas J. Hope, Xiaoying Shen, Linda Kalilani, Genevieve G. Fouda, Georgia D. Tomaras, Sallie R. Permar

**Affiliations:** a Duke Human Vaccine Institute, Duke University, Durham, North Carolina, USA; b Department of Molecular Genetics and Microbiology, Duke University, Durham, North Carolina, USA; c Department of Surgery, Duke University, Durham, North Carolina, USA; d Department of Pediatrics, Duke University, Durham, North Carolina, USA; e Department of Immunology, Duke University, Durham, North Carolina, USA; f Department of Cell and Molecular Biology, Feinberg School of Medicine, Northwestern University, Chicago, Illinois, USA; g College of Medicine, University of Malawi, Blantyre, Malawi; Emory University

**Keywords:** HIV-1, IgA, effector functions, mucosal immunity

## Abstract

The humoral response to invading mucosal pathogens comprises multiple antibody isotypes derived from systemic and mucosal compartments. To understand the contribution of each antibody isotype/source to the mucosal humoral response, parallel investigation of the specificities and functions of antibodies within and across isotypes and compartments is required. The role of IgA against HIV-1 is complex, with studies supporting a protective role as well as a role for serum IgA in blocking effector functions. Thus, we explored the fine specificity and function of IgA in both plasma and mucosal secretions important to infant HIV-1 infection, i.e., breast milk. IgA and IgG were isolated from milk and plasma from 20 HIV-1-infected lactating Malawian women. HIV-1 binding specificities, neutralization potency, inhibition of virus-epithelial cell binding, and antibody-mediated phagocytosis were measured. Fine-specificity mapping showed IgA and IgG responses to multiple HIV-1 Env epitopes, including conformational V1/V2 and linear V2, V3, and constant region 5 (C5). Env IgA was heterogeneous between the milk and systemic compartments (Env IgA, τ = 0.00 to 0.63, *P* = 0.0046 to 1.00). Furthermore, IgA and IgG appeared compartmentalized as there was a lack of correlation between the specificities of Env-specific IgA and IgG (in milk, τ = −0.07 to 0.26, *P* = 0.35 to 0.83). IgA and IgG also differed in functions: while neutralization and phagocytosis were consistently mediated by milk and plasma IgG, they were rarely detected in IgA from both milk and plasma. Understanding the ontogeny of the divergent IgG and IgA antigen specificity repertoires and their effects on antibody function will inform vaccination approaches targeted toward mucosal pathogens.

**IMPORTANCE** Antibodies within the mucosa are part of the first line of defense against mucosal pathogens. Evaluating mucosal antibody isotypes, specificities, and antiviral functions in relationship to the systemic antibody profile can provide insights into whether the antibody response is coordinated in response to mucosal pathogens. In a natural immunity cohort of HIV-infected lactating women, we mapped the fine specificity and function of IgA in breast milk and plasma and compared these with the autologous IgG responses. Antigen specificities and functions differed between IgG and IgA, with antiviral functions (neutralization and phagocytosis) predominantly mediated by the IgG fraction in both milk and plasma. Furthermore, the specificity of milk IgA differed from that of systemic IgA. Our data suggest that milk IgA and systemic IgA should be separately examined as potential correlates of risk. Preventive vaccines may need to employ different strategies to elicit functional antiviral immunity by both antibody isotypes in the mucosa.

## Abstract (precis)

Antibodies within the mucosa are part of the first line of defense against mucosal pathogens. Evaluating mucosal antibody isotypes, specificities, and antiviral functions in relationship to the systemic antibody profile can provide insights into whether the antibody response is coordinated in response to mucosal pathogens. In a natural immunity cohort of HIV-infected lactating women, we mapped the fine specificity and function of IgA in breast milk and plasma and compared these with the autologous IgG responses. Antigen specificities and functions differed between IgG and IgA, with antiviral functions (neutralization and phagocytosis) predominantly mediated by the IgG fraction in both milk and plasma. Furthermore, the specificity of milk IgA differed from that of systemic IgA. Our data suggest that milk IgA and systemic IgA should be separately examined as potential correlates of risk. Preventive vaccines may need to employ different strategies to elicit functional antiviral immunity by both antibody isotypes in the mucosa.

## INTRODUCTION

There is increasing appreciation that the mucosal antibody response may be key for protection against some mucosal pathogens, including influenza virus (1) and rotavirus (2). Similarly, humoral responses against HIV-1 at mucosal surfaces may be important for human immunodeficiency virus (HIV) prevention as approximately 90% of HIV transmissions occur at mucosal sites (3). While assessment of immune responses generally focuses on the systemic compartment, the mucosal and systemic antibody responses can be distinctly regulated (4), and such differential stimulation of mucosal and systemic humoral responses has been shown in several human vaccine studies, with differences depending on factors including the route of immunization, immunogen, and adjuvant (5–8). It is therefore necessary to evaluate the relationship between mucosal and systemic antibody responses.

While IgG is more abundant than IgA in the plasma, IgA is more abundant than IgG at most mucosal surfaces. IgA comprises two subclasses, IgA1 and IgA2, both of which are capable of existing in monomeric and dimeric forms. Dimeric IgA (dIgA) is capable of associating with the polymeric Ig receptor (pIgR), which allows transcytosis of dimeric IgA across the epithelial lining into mucosal surfaces. pIgR is subsequently cleaved from the dIgA molecule, leaving behind an associated secretory component; together, these are known as secretory IgA (sIgA). The secretory component occupies the site at which FcαRI binds (9); thus, sIgA may be restricted to particular Fc-mediated activities (10, 11), which may be mediated by other receptors such as Mac-1 (12, 13). IgA in the plasma comprises mostly monomeric IgA (mIgA), whereas IgA in the mucosal fluids is comprised mostly of sIgA, with a small fraction of monomeric or dimeric IgA. Investigating the relationship between IgG and IgA in terms of both antigen specificities and functions will facilitate the discovery of mechanistic immune correlates of protection and contribute toward vaccine designs aimed at the targeting of particular isotype and antigen profiles.

The challenges of examining antibody responses in external mucosal secretions include variable amounts of fluid and immunoglobulin able to be collected, differing methods of collection, various hormone levels, and/or menstrual cycle timing in study participants. Additionally, protease sensitivity has hindered investigations of the ability of mucosal antibodies to mediate antimicrobial functions (14). These challenges, coupled with low Env-specific mucosal IgA levels during HIV-1 infection and after vaccination, have led to a poorly understood role of mucosal antibodies in defense against HIV-1, impeding our ability to determine whether mucosal responses need to be targeted by HIV-1 vaccine candidates.

Breast milk offers a unique opportunity to study mucosal antibodies. In contrast to other mucosal secretions such as tears, saliva, and genital secretions, large amounts of this high-antibody-containing secretion can be easily collected in lactating women. The IgA response in breast milk has been reported to reflect that of distant mucosal sites due to the homing of IgA-expressing memory B cells and plasma cells from the gastrointestinal tract to the mammary tissue via the gut-mammar*y* axis (15–17). Breast milk is also advantageous for the study of sIgA because the concentration of sIgA remains high throughout lactation (18), unlike total sIgA levels in the female genital tract, which differ greatly throughout the menstrual cycle (14, 19–21). Investigating breast milk antibodies in parallel with systemic antibodies can, thus, shed light on the relationship between antibody specificities between the mucosal and systemic IgG, IgA, and sIgA compartments.

We recently described an association between the magnitude of breast milk total and secretory IgA (sIgA) responses against HIV-1 gp140 and reduced risk of postnatal HIV-1 transmission in the Malawian Breastfeeding, Antiretrovirals, and Nutrition (BAN) study (22). Yet HIV-1 Env-specific IgA responses are generally low in mucosal compartments of HIV-infected individuals (23–27). Importantly, Ruprecht et al. have demonstrated that mucosally applied Env-specific dimeric IgA1 (28) or mucosally applied dimeric IgA2 in combination with systemic IgG1 (16) can provide protection against high-dose intrarectal simian-human immunodeficiency virus (SHIV) challenge. In contrast, in the adult RV144 vaccine trial, which showed moderate efficacy in prevention of mucosal HIV-1 acquisition, certain specificities of plasma HIV-1 envelope (Env)-specific gp120 and gp140 IgA responses correlated with increased HIV-1 risk (i.e., decreased vaccine efficacy) (29), potentially due to constant region 1 (C1)-C2-specific IgA antibodies blocking corresponding IgG antibody-dependent cellular cytotoxicity (ADCC) function (30). These results suggest differences in the ability of mucosal and systemic HIV-1 Env-specific IgA and IgG antibodies to modulate HIV-1 acquisition. Defining differences in specificities and/or functions may help in understanding the distinct association of mucosal and systemic IgA and IgG with HIV acquisition risk in the context of natural vertical transmission and will also aid the discovery of mechanistic correlates of HIV-1 protection.

Investigating the specificity and functions of HIV-1 Env-specific antibodies from breast milk is critical to further define its potential protective mechanisms in breast milk HIV-1 transmission and improve our ability to target these responses in the development of a vaccine to prevent postnatal HIV-1 transmission. Vaccines to eliminate postnatal HIV-1 transmission are necessary in developing areas of high HIV-1 prevalence in particular, where breastfeeding is responsible for one-third to one-half of vertical HIV-1 infections (25, 31). Additionally, formula feeding in these regions is generally not recommended due to the high incidence of respiratory and diarrheal diseases in nonbreastfed infants (32).

The goal of this study was to define the relationship between the specificity and function of mucosal and systemic IgG and IgA in the context of HIV-1 infection. We purified polyclonal IgA and IgG from breast milk and plasma of a cohort of 20 HIV-infected lactating Malawian women and investigated the fine Env antigen specificity of their anti-HIV-1 antibody responses, as well as anti-HIV-1 antibody functions, including neutralization, inhibition of epithelial cell binding, and phagocytosis. Defining the IgG and IgA specificities that are associated with antiviral functions in mucosal and systemic compartments is key to rationally targeting specific responses at the portal of HIV-1 entry.

## RESULTS

### Heterogeneity between HIV-1 Env antigen specificities of milk and plasma IgAs.

**FIG 1:**
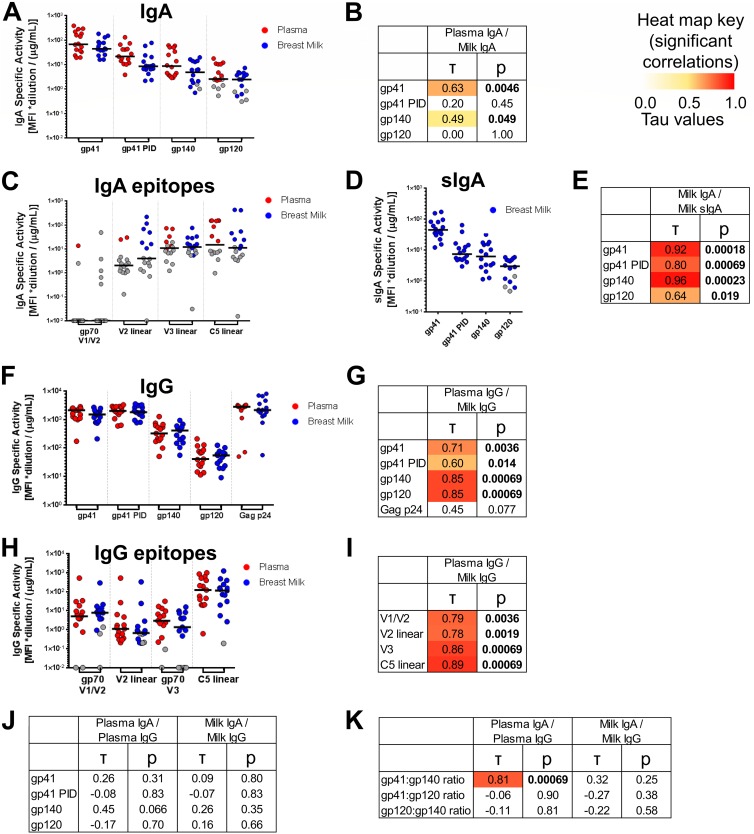
Env antigen specificities of milk IgG and IgA and plasma and milk IgAs do not correlate, while milk and plasma IgG Env antigen-specificities are strongly correlated. (A, C, D, F and H) For 16 HIV-1^+^ lactating women, milk and plasma samples were obtained, and IgA and IgG were purified from both sample types. For IgA (A) and sIgA (D), binding scores to gp41, gp41 PID, gp140, and gp120 antigens are shown (see Materials and Methods for antigens and calculation). For IgA, scores for binding to gp70 V1/V2, linear V2, linear V3, and linear C5 antigen subspecificities are also shown (C). For IgG, scores are shown for binding to Env gp41, gp41 PID, gp140, and gp120, as well as Gag p24 antigens (F), and to gp70 V1/V2, linear V2, gp70 V3, and linear C5 antigen subspecificities (H). Background-subtracted MFI values below 0 are shown with a value of 0.01. (B, E, G, I, J and K) Correlations between compartments for each antigen specificity or antigen specificity ratio. Kendall’s tau values and corresponding corrected *P* values (see Materials and Methods) are reported. Boldface *P* values indicate significant correlations at a *P* value of <0.05, with corresponding tau values color coded on a scale of 0 to 1.

We first compared the antigen specificities of breast milk and plasma IgAs. IgA was purified from corresponding breast milk and plasma samples of 20 HIV-1-infected Malawian women from the previously described CHAVI 009 cohort (33). IgA purification from breast milk yielded a median of 1.49 mg IgA/ml of breast milk (range, 0.05 to 5.87 mg/ml), whereas IgA purification from plasma yielded a median of 0.65 mg IgA/ml of plasma (range, 0.23 to 1.09 mg/ml), similar to results of our previous study (33). HIV-1 specific activity (SA) for antigens or antigen groups was calculated by taking the mean fluorescence intensity (MFI) from the multiplex binding assay, multiplying by dilution, and dividing by purified Ig concentration in micrograms/milliliter (see Materials and Methods). As previously reported in studies of natural HIV-1 infection (34), conformational and linear gp41 responses represented the dominant IgA Env antigen specificity, followed by gp140 and gp120 responses (gp41 binding in milk: 100% positive, median SA of 43.9; gp41 principal immunodominant domain [gp41 PID] in milk: 100% positive, median SA of 8.41; gp140 binding in milk: 86% positive, median SA of 4.83; gp120 binding in milk: 75% positive, median SA of 2.55) (Fig. 1A). To map the antigen specificity of the IgA HIV-specific response in more detail than in previous studies (35), IgA binding was tested against a panel of conformational (V1/V2) and linear (V2, V3, and C5) Env antigens. We observed at least one participant with a plasma or milk IgA binding response to all antigens tested, albeit with low positivity rates that could be due to levels below the limit of detection of the assay (Fig. 1C). In fact, in a few participants, subdominant IgA responses (i.e., V1/V2 relative to gp140, gp120 relative to gp140, or C5 relative to gp41) were elevated above ratios seen for the IgG compartment (described below), indicating that the IgA response is not narrower in antigen specificity than the IgG response.

**FIG 2:**
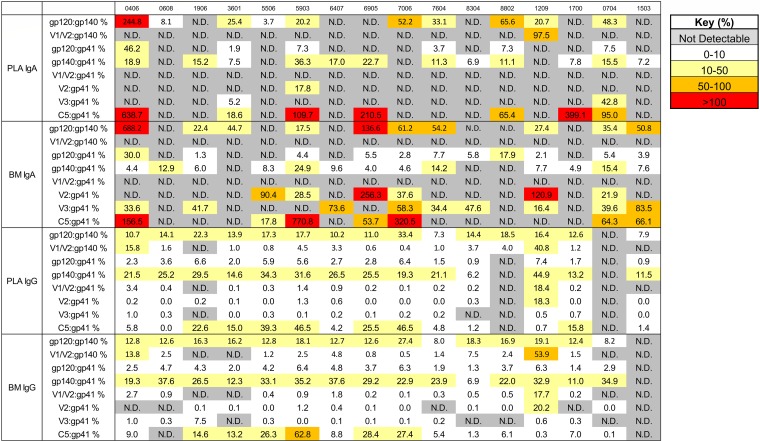
Heterogeneity of the Env antigen specificities of breast milk (BM) and plasma (PLA) IgGa and IgAa between individuals. For each of 16 HIV-1^+^ individuals, the relationships between selected pairwise binding scores are displayed as percentages. N.D. indicates that either one of the pair of values was a missing value or exceeded the limit of quantitation (<100 MFI or >23,000 MFI).

**FIG 3:**
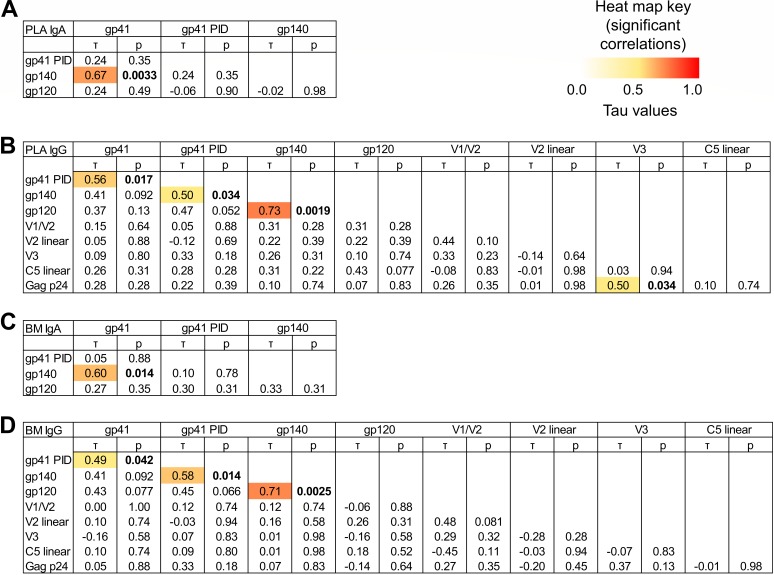
Env antigen-specific IgA and IgG responses in milk and plasma seldom correlate with other Env antigen-specific responses in HIV-infected lactating women. To determine whether immune responses to the various HIV-1 antigens were associated with each other, correlations between binding scores for selected antigen specificities were tested for plasma IgA (A), plasma IgG (B), breast milk (BM) IgA (C), and breast milk IgG (D). Kendall’s tau values and corresponding corrected *P* values (see Materials and Methods) are reported. Boldface *P* values indicate significant correlations at a *P* value of <0.05, with corresponding tau values color coded on a scale of 0 to 1.

Despite these trends in antigen specificity profile, heterogeneity in antigen specificity dominance of IgA in both breast milk and plasma was observed between individuals. For instance, among participants with positive responses to both gp120 and gp140 antigens, the ratio of milk gp120/gp140 activity ranged from 0.18 to 6.88, and the plasma gp120/gp140 ratio ranged from 0.04 to 2.45 (Fig. 2). In support of such heterogeneity, few significant correlations were observed between gp120, gp140, and gp41 IgA activities across individuals (Fig. 3A and C). Thus, while mostly gp41- and gp140-dominant responses are observed, interindividual heterogeneity in the anti-Env IgA response occurs in both plasma and breast milk of HIV-1-infected individuals.

We next compared the IgA response specificities in milk and plasma of each participant. Most women had slightly lower milk IgA specific activity to specific antigens than in plasma, with a milk/plasma IgA ratio lower than 1 (for gp41, average ratio = 0.76, *P* = 0.017 [specific activity magnitude comparison by Wilcoxon signed rank test]; for gp41 PID, ratio = 0.60, *P* = 0.023; for gp140, ratio = 0.50, *P* = 0.010; for gp120, ratio = 0.86, *P* = 0.23) although total IgA activity remains higher in milk than in plasma since total IgA concentration was 0.5 to 1 log higher in milk (33). To further understand the potential antigen specificity differences between mucosal and systemic IgA, we investigated if there was a correlation between Env-specific IgA activity in milk and plasma. Modest correlations were observed for gp140 (τ = 0.49, *P* = 0.049) and gp41 antigens (τ = 0.63, *P* = 0.0046); however, there was no correlation between compartments for gp120 IgA (τ = 0.00, *P* = 1.00) (Fig. 1B). These results suggest that there is differential compartmentalization of certain specificities of HIV-1 Env IgA responses between milk and plasma.

### The majority of Env-specific IgA in breast milk exists in the form of sIgA.

Milk IgA is known to exist largely in the form of sIgA. We sought to confirm whether this was true also of the HIV-1 Env-specific IgA response. Thus, sIgA responses were assessed against HIV-1 Env antigens. As expected, very few positive sIgA responses were seen for plasma IgA, whereas Env-specific sIgA was detected in milk sIgA (Fig. 1D). Milk gp41 IgA and sIgA responses were compared via normalization against a standard curve of 7B2 IgA and sIgA, respectively. sIgA comprised at least 80% of total IgA in 100% (gp41 PID), 83% (gp41 clade B), and 63% (gp41 clade C) of participants with quantifiable sIgA and IgA antigen-specific responses, showing that the majority of total Env-specific IgA exists in the form of sIgA. To assess the similarity of the milk IgA and sIgA response epitope specificities across other antigen specificities, we examined the relationship between the milk IgA and sIgA compartments for gp41, gp41 PID, gp140, and gp120 antigen specificities. In support of the similarities between milk IgA and sIgA compartments, the milk IgA and sIgA responses were strongly correlated (Fig. 1E). These results suggest that the milk sIgA antigen specificity profile is highly related to the total milk IgA antigen specificity within an individual, consistent with the dominance of sIgA in the milk Env-specific IgA response.

### Correlation of HIV-1 Env antigen specificity between breast milk and plasma IgGs.

To further understand the antibody composition within mucosal secretions, IgG was also isolated from milk and plasma in parallel from the same participants. The antigen specificities of the breast milk and plasma IgG preparations were assessed against the same multiclade panel of HIV-1 antigens (Fig. 1F and H). As with IgA, gp41 responses represented the dominant IgG Env antigen specificity, followed by gp140 and gp120 responses (gp41 binding in milk IgG: 100% positive, median SA of 1,510; gp41 PID binding in milk IgG: 100% positive, median SA of 1,799; gp140 binding in milk IgG: 100% positive, median SA of 409; gp120 binding in milk IgG: 100% positive, median SA of 54.9) (Fig. 1F). However, IgG responses to other conformational and linear Env antigens were more prevalent than those in IgA (conformational V1/V2 binding in milk IgG: 80% positive, median SA of 7.76; linear V2 peptide binding in milk IgG: 73% positive, median SA of 0.662; V3 binding in milk IgG: 73% positive, median SA of 1.36; linear C5.2 peptide binding in milk IgG: 93% positive, median SA of 114) (Fig. 1H).

Similar to what was seen with IgA, heterogeneity in antigen specificity dominance in IgG responses was observed between individuals. For instance, among participants with positive responses to all antigen categories, ratio of activity (as a percentage) of milk IgG V1/V2 to gp140 had an ∼2-log range from 0.5% to 53.9% (Fig. 2). To further examine the heterogeneity in antigen specificity dominance across individuals, we examined the pairwise correlation between IgG response magnitude to each antigen specificity (gp41, gp41 PID, gp140, gp120, gp70 V1/V2, V2 linear, gp70 V3, C5 linear, and p24). Few correlations were consistently observed across antigen specificities: with the exceptions of pairwise correlations found between gp120/gp140, gp140/gp41 PID, and gp41/gp41 PID responses, no other linear or conformational antigen specificities consistently showed pairwise correlation (Fig. 3B and D). Thus, as with IgA, interindividual heterogeneity in the anti-Env IgG response occurs in both plasma and breast milk of HIV-1-infected individuals in this cohort.

To compare the mucosal and systemic IgG compartments, we compared the specificity of the IgG responses in milk and plasma of each participant. IgG specific activities against most Env glycoproteins were similar between milk and plasma, with a milk/plasma IgG ratio close to 1 (for gp41, average ratio = 0.84, *P* = 0.0028 [specific activity magnitude comparison by Wilcoxon signed rank test]; for gp41 PID, ratio = 0.99, *P* = 0.64; for gp140, ratio = 0.86, *P* = 0.084; for gp120, ratio = 0.88, *P* = 0.18; for gp70 V1/V2, ratio = 1.07, *P* = 0.61; V2 linear, ratio = 0.61, *P* = 0.0019; for gp70 V3, ratio = 0.58, *P* = 0.0097; for C5 linear, ratio = 0.82, *P* = 0.28) (Fig. 1F and H) although it should be noted that total IgG activity is higher in plasma across all antigens since plasma contains a 2-log-higher concentration of IgG than breast milk (33). To further understand the potential antigen specificity differences between mucosal and systemic IgG, we investigated if there was a correlation between Env-specific IgG activities in milk and plasma. In fact, there were strong correlations for each antigen specificity across milk and plasma IgG compartments (Fig. 1G and I). These results confirm previous findings that the IgG antigen specificity profile within individuals is strongly conserved across plasma and milk compartments, which is distinct from the only marginal correlations in specificities between plasma and breast milk IgA responses (33).

### HIV-1 Env antigen specificities differ between plasma and milk IgG and IgA.

To understand the relationship between the IgG and IgA antigen specificity profiles, we investigated if the IgG antigen specificity profile was correlated with the IgA antigen specificity profile. There was no correlation between milk IgA and milk IgG specific activities; neither was there correlation between plasma IgA and IgG (Fig. 1J). We further tested if the fraction of activity directed toward each antigen was conserved across isotype by examining whether the ratio of antigen specificities (gp41/gp140, gp41/gp120, and gp120/gp140) was correlated between isotypes (Fig. 1K). In milk, no correlations were found between IgA and IgG. In plasma, no correlations were found for the gp41/gp120 ratio and gp120/gp140 ratio. While a correlation between plasma IgA and IgG was found for the gp41/gp140 ratio (τ = 0.81, *P* = 0.00069), further investigation demonstrated that the magnitudes of the gp140/gp41 ratios were significantly different between IgG and IgA, with the IgG response having a higher gp140/gp41 ratio than IgA (average ratios of 0.23 and 0.16, respectively; *P* = 0.0077). Thus, the antigen specificity profiles of milk and plasma IgGs and IgAs, measured either in terms of specific activity of each antigen or in terms of the fraction of total activity directed toward each antigen, are not conserved across isotype. This suggests that the antigen specificities of plasma and milk IgG and IgA are compartmentalized.

### Breast milk and plasma IgGs, but rarely IgAs, mediate HIV-1 neutralization function.

**FIG 4:**
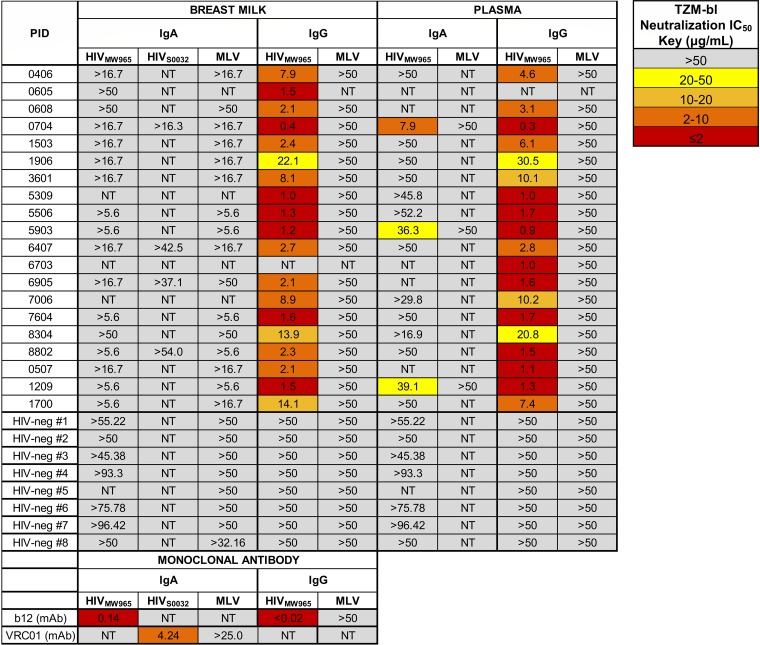
Milk and plasma IgG, but not IgA, mediate tier 1 HIV_MW965_ neutralization. To identify HIV-1 neutralization function in antibody fractions of milk and plasma, we determined the neutralization IC_50_ in TZM-bl cells for IgA and IgG antibodies isolated from milk and plasma samples of 20 participants. HIV-specific neutralization activities against the clade C tier 1 HIV variants MW965 (and also S0032 for selected samples) are shown, as well as nonspecific antiviral neutralization activities against murine leukemia virus (MLV). Eight HIV-negative controls are also shown, as well as positive-control recombinant monoclonal antibodies (b12 and VRC01, CD4 binding site broadly neutralizing antibodies) in IgA and IgG backbones. No milk IgA samples neutralized HIV-1_MW965_ or HIV-1_S0032_, whereas three plasma IgA samples had detectable neutralization.

The ability of purified IgG/IgA from milk and plasma to mediate virus neutralization was assessed via a TZM-bl cell-based assay against a clade C neutralization-sensitive tier 1 virus, HIV-1_MW965_. Milk IgA had no HIV-specific neutralization above background levels although sensitivity in the assay was reduced due to cell toxicity at high antibody concentrations (Fig. 4). In plasma IgA, 20% of samples showed HIV-specific neutralization. To confirm these results, we examined the ability of four milk IgA samples with adequate purified IgA for additional testing to mediate neutralization against another clade C virus, HIV-1_S0032_. No specific neutralization was seen for milk IgA against this virus strain as well. Therefore, in the setting of HIV-1 infection, IgA does not appear to have strong HIV-specific functional activity for neutralization (at least against the clade-matched HIV-1_MW965_ and HIV-1_S0032_ strains tested) in either milk or plasma compartments although IgA-mediated neutralization may occur rarely in the plasma compartment. In contrast, all milk and plasma IgG preparations tested had detectable neutralization activities, with HIV-1_MW965_ neutralization 50% inhibitory concentration (IC_50_) values comparable (within 3-fold) between milk and plasma from the same participant, consistent with our previous observations (33). Thus, in this cohort, IgG appears to be more potent than IgA in neutralization activity, yet neutralizing function is conserved across milk and systemic compartments.

**FIG 5:**
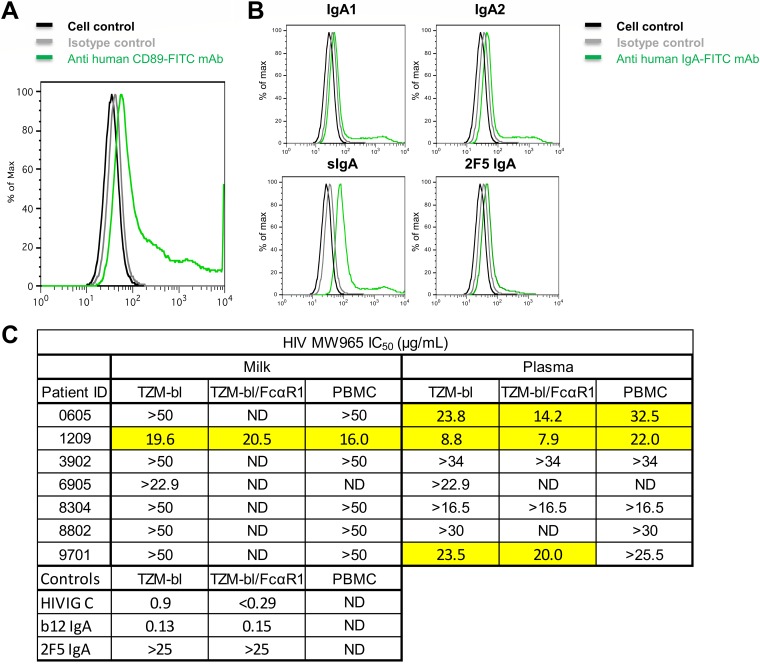
IgA-mediated tier 1 HIV-1 neutralization is not enhanced in FcαRI-expressing cells or peripheral blood mononuclear cells. (A) To gain insight into the role of CD89 in HIV infectivity and neutralization, the cDNAs for the human FcαRI and the γ chain of the FcεR were transduced into TZM-bl cells using lentiviral constructs. Specific monoclonal antibodies and flow cytometry detected surface expression of FcαRI on this new TZM-bl cell line. (B) Flow cytometry diagrams show the capability of the TZM-bl/FcαRI cell line to bind to human monomeric IgA1 and monomeric IgA2, human colostrum secretory IgA, and monoclonal 2F5 IgA. (C) To determine whether neutralization activity is enhanced in the presence of Fc alpha receptor 1 (FcαRI), we examined the neutralization IC_50_ using TZM-bl cells transduced with FcαRI and also in human peripheral blood mononuclear cells (PBMCs). Milk and plasma IgAs were tested for six participants with a variety of neutralization phenotypes in TZM-bl cells. The positive controls HIVIG (clade C) and b12 IgA MAb and the negative-control 2F5 MAb (broadly neutralizing, but does not neutralize MW965) were also tested. ND, not done for the indicated sample. Neutralization was not enhanced in either FcαRI-transduced TZM-bl cells or in peripheral blood mononuclear cells.

To examine if the expression of FcαRI on the target cells would improve IgA-mediated neutralization, the IgA isolated from five plasma samples and one milk sample were tested for neutralization potency against HIV-1_MW965_ in TZM-bl cells engineered to express FcαRI (36) (Fig. 5A). These TZM-bl/FcαRI cells are capable of binding to IgA1, IgA2, and sIgA (Fig. 5B). Such FcR binding between IgG and FcγRI has been shown to be capable of enhancing neutralization by particular specificities of IgG (37) though the mechanism remains unclear (independent of phagocytosis) (38). However, samples with undetectable neutralization potency in TZM-bl cells also had undetectable neutralization potency in TZM-bl/FcαRI cells, while samples with detectable neutralization potency in TZM-bl cells had neutralization activity with similar IC_50_s (within 3-fold) in TZM-bl/FcαRI cells (Fig. 5C). Thus, in this cohort, presence of the FcαRI receptor does not enhance the neutralizing function of Env-specific IgA.

### Breast milk and plasma IgAs may inhibit epithelial cell binding.

**FIG 6:**
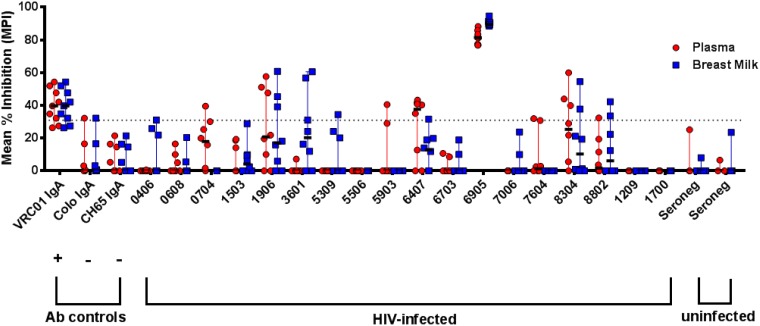
Breast milk and plasma IgAs may inhibit C.1086 HIV-1 virion binding to epithelial cells. Inhibition of binding to the colonic epithelial cell line HT-29 was assessed using the tier 2 clade C virus HIV-1 C.1086, with 7 to 12 replicates performed over three independent experiments. VRC01 IgA was used as a positive control, and HIV-negative colostrum IgA and anti-influenza virus CH65 IgA were used as negative controls. A dotted line indicates the mean percent inhibition (MPI) cutoff of 31%, calculated as 2 standard deviations plus the MPI of anti-influenza virus hemagglutinin MAb CH65 IgA relative to that of the no-antibody condition.

We next assessed the ability of milk and plasma IgA and IgG antibodies to inhibit epithelial cell binding of infectious virions, an important first step in blocking virus transmission at a mucosal surface. Interestingly, both milk and plasma purified IgA preparations isolated from one participant inhibited epithelial cell binding against clade C virus HIV-1 Ce.1086 (mean percent inhibition, 91% and 84%, respectively) (Fig. 6). None of the other milk or plasma IgA or IgG preparations consistently inhibited epithelial cell binding, potentially due to a low sensitivity for this assay type, although, it should be noted that several individual data points for multiple participants (notably plasma IgA for participant 6407) were above the positivity cutoff. Therefore, in our cohort of HIV-1-infected persons, both milk and systemic IgA may mediate epithelial cell binding inhibition.

### Breast milk and plasma IgGs, but rarely IgAs, mediate HIV-1 phagocytosis function.

**FIG 7:**
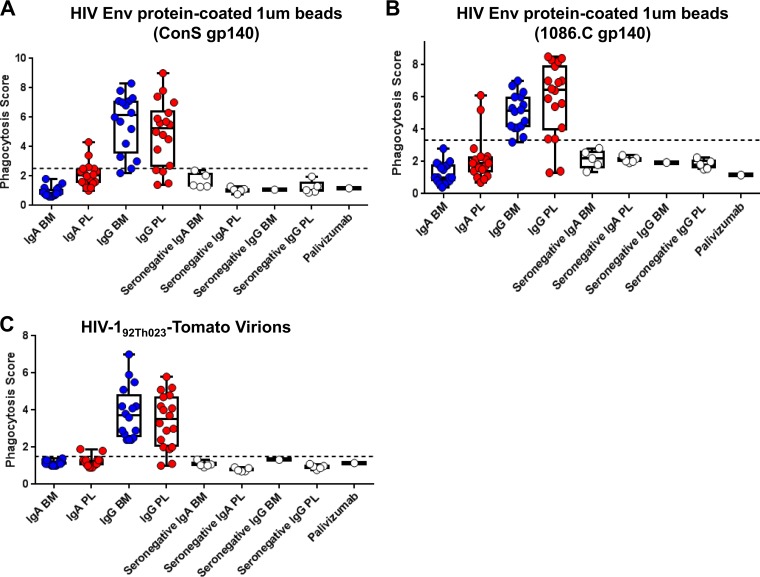
Breast milk and plasma IgGs, but not IgAs, mediate phagocytosis of HIV-1 virions and Env-coated beads. To determine the phagocytosis function in antibody fractions of breast milk (BM) and plasma (PL), we tested IgA and IgG from milk and plasma of 16 HIV^+^ women for phagocytosis of beads coated with HIV-1 Env ConS gp140 (A), beads coated with HIV-1 Env 1086.C gp140 (B), and fully infectious fluorescent HIV-1 virions (HIV-1_92Th023_-Tomato) (C). Antibodies from an additional five HIV-negative women were also tested as negative controls, as well as the anti-respiratory syncytial virus antibody palivizumab. The black dotted line indicates the positivity cutoff, determined using the mean +3 standard deviations of values of the negative controls used in the assay.

**FIG 8:**
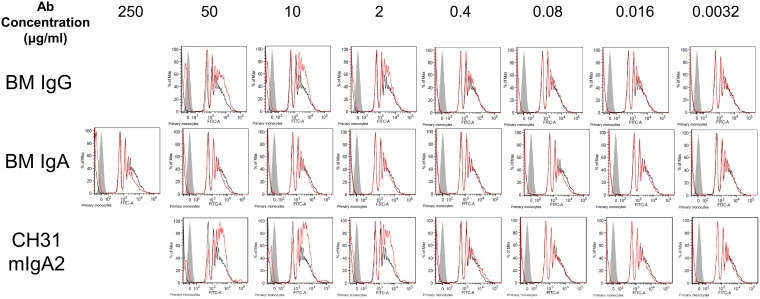
Lack of detectable milk IgA-mediated phagocytosis despite increasing IgA concentration. To determine if lack of milk IgA phagocytosis activity was due to lower HIV-1 specific activity in IgA than that in IgG, higher concentrations of IgA and IgG were purified from the milk of an HIV-positive lactating woman recruited in the United states. Breast milk (BM) IgG, breast milk IgA, and a control antibody, CH31 mIgA2, were tested for phagocytosis of HIV ConS gp140-coated beads at 5-fold dilutions as indicated, and flow cytometry diagrams indicative of the phagocytosis results are shown. The red traces indicate sample antibody setup while black traces indicate the no-antibody control setup, and gray fill indicates the no-target control setup. Antibody-mediated phagocytosis is indicated by a greater area under the curve for the red trace than that for the black trace. Milk IgG and CH31 mIgA2 showed antibody-mediated phagocytosis activity down to 2 μg/ml, while milk IgA showed no antibody-mediated phagocytosis activity even at 250 μg/ml.

We next assessed the ability of milk and plasma IgA and IgG antibodies to mediate phagocytosis. Phagocytosis of a consensus target (ConS gp140) as well as a clade-matched target (1086.C gp140) was measured by coating the respective Env proteins on labeled beads and then measuring the uptake of labeled beads by human primary monocytes in the presence or absence of sample antibody (Fig. 7). Against ConS gp140-coated beads, 0% of milk IgA and 19% of plasma IgA samples showed detectable phagocytosis activity, while 86% of milk IgG and 86% of plasma IgG samples showed detectable phagocytosis activity (Fig. 7A). Results in 1086.C gp140-coated beads were similar (Fig. 7B). Rarity of IgA-mediated phagocytosis responses was not due to insensitivity of the monocytes to IgA since a positive-control antibody, CH31 mIgA2, was capable of mediating phagocytosis (Fig. 8), in accordance with our previous reports of IgA-mediated phagocytosis activity in human primary monocytes (39). Thus, the IgG compartment is more potent than the IgA compartment in phagocytosis activity in this cohort. While functionality is conserved across milk and plasma for IgG, phagocytosis activity is rarely detected in plasma IgA and is absent in milk IgA.

To see if these results extended to antibody-mediated phagocytosis of infectious virions, infectious fluorescent HIV-1_92TH023_-Tomato virions (clade A/E) were also tested as phagocytosis targets (Fig. 7C). Zero percent of milk and 13% of plasma IgA samples showed detectable phagocytosis activity, while 100% of milk and plasma IgG samples showed detectable phagocytosis activity. Thus, in this cohort, the plasma and milk IgG samples were also more potent than the IgA samples for virion phagocytosis.

All antibodies were tested at 50 μg/ml total antibody concentration regardless of HIV-1-specific activity. To confirm that the observed results were not due to lower HIV-1-specific activity in the IgA assays, titrated HIV-positive (HIV^+^) breast milk IgA and IgG were tested for phagocytosis activity against ConS gp140-coated beads (Fig. 8). HIV^+^ breast milk IgA did not mediate phagocytic activity against ConS gp140-coated beads even at 250 μg/ml, while HIV^+^ breast milk IgG mediated phagocytic activity against ConS gp140-coated beads down to 2 μg/ml. Thus, the reduced milk IgA phagocytic function observed was not due to lower IgA HIV-specific activity.

### IgG neutralization and phagocytosis functions are moderately correlated.

**FIG 9:**
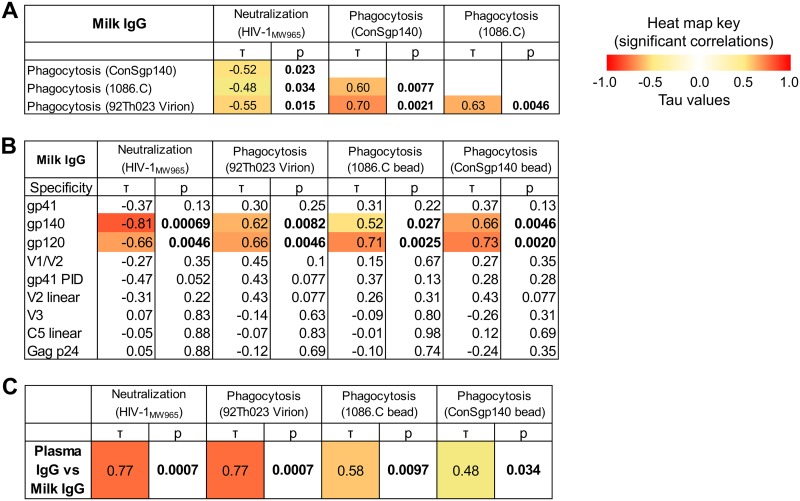
Functional IgG activity in milk and plasma are correlated and linked to antibodies targeting gp140 and gp120. (A) To determine if the functional activity of anti-HIV-1 antibodies in milk were associated with each other, correlations were tested between neutralization (HIV-1_MW965_) and phagocytosis (ConS gp140 beads, 1086.C beads, and 92Th023 virions) for milk IgG. Kendall’s tau values and corresponding corrected *P* values (see Materials and Methods) are reported. Boldface *P* values indicate significant correlations at a *P* value of <0.05, with corresponding absolute tau values color coded on a scale of 0 to 1. (B) To determine candidate antigen specificities that could be targeted for functional activity by milk IgG antibodies, correlations were tested between antigen-specific binding and functional activity. (C) To identify whether functional activity was conserved across milk and plasma antibodies, correlations were tested between milk and plasma IgGs for each antibody function (neutralization and phagocytosis).

**FIG 10:**
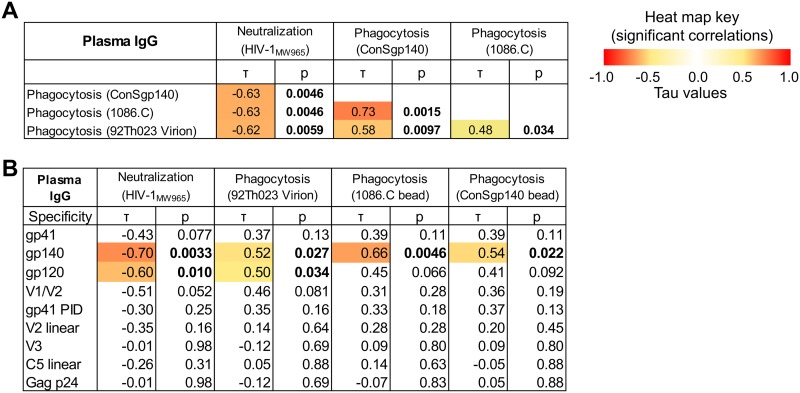
Correlations between antibody functions and epitope specificity in plasma IgG. (A) To determine if the functional activity of anti-HIV-1 antibodies in milk were associated with each other, correlations were tested between neutralization (HIVMW965) and phagocytosis (ConS gp140 beads, 1086.C beads, 92Th023 virions) for plasma IgG. Kendall’s tau values and corresponding corrected *P* values (see Materials and Methods) are reported. Boldface *P* values indicate significant correlations at a *P* value of <0.05, with corresponding absolute tau values color coded on a scale of 0 to 1. (B) To determine candidate antigen specificities that could be targeted for functional activity by plasma IgG antibodies, correlations were tested between antigen-specific binding and functional activity.

To assess if antibody functions are related, neutralization and antibody-mediated phagocytosis activities were tested for correlation. IgA was excluded from this analysis due to the low IgA response rate for both neutralization and phagocytosis. Milk IgG-mediated neutralization and phagocytosis functions were modestly related (lower IC_50_ is more potent neutralization) (−0.55 < τ < −0.48), and phagocytosis functions were correlated across both virus and bead targets (0.60 < τ < 0.70) (Fig. 9A). Similar correlations were found between plasma IgG functions (Fig. 10A). In addition, milk and plasma functional activities were correlated for each function (0.48 < τ < 0.77) (Fig. 9C). Thus, IgG functional activity in both plasma and milk are conserved.

### IgG gp140 and gp120 antigen specificities are associated with neutralization and phagocytosis functions.

We sought to investigate the antigen specificities that contribute to functional activity against virions (i.e., virion phagocytosis and neutralization) in plasma and mucosal compartments. To do so, milk and plasma IgG binding responses to Env gp120, gp140, gp41, V1/V2, V3, and C5 antigens and Gag p24 antigen were examined for correlation with milk and plasma IgG phagocytosis and neutralization functions, respectively (Fig. 9B and 10B). For milk IgG, both neutralization and phagocytosis functions correlated with gp140 and gp120 binding responses but not with gp41, gp41 PID, V1/V2, V2 linear, V3, C5.2 linear, and p24 responses. The antigen specificities associated with plasma IgG function were similar to those found in milk (Fig. 10B). Thus, regardless of compartment, IgG antibodies to conformational Env gp140 and gp120 are associated with neutralization and phagocytosis functions, while antibodies to Gag p24 and the linear epitope specificities in Env tested here (i.e., V2, V3, and C5) are not associated with antibody effector functions.

## DISCUSSION

HIV-1 is predominantly a mucosal pathogen and interacts with the humoral immune response first at the mucosal site of entry. However, most studies on the humoral immune response against HIV-1 have focused on understanding the systemic antibody response, in part due to limited mucosal sample availability. We hypothesized that the Env-specific IgA humoral immune responses would be distinct in specificity and function between breast milk and plasma as well as differ from those of IgG antibodies. We probed these relationships on two levels: antigen specificity and function. Analyses of antigen specificity and antiviral functions provide insights on the relatedness of anatomical compartments in terms of antibody and B cell trafficking. Studies that define unique features of IgG and IgA antigen specificities and functions between mucosal and systemic compartments can inform further studies to modulate the immune response toward protective immunity by vaccination.

To define the relationship between mucosal and systemic humoral responses in the context of HIV-1 infection, we sought to compare milk and plasma antibodies from HIV-1-infected lactating women as representatives of mucosal and systemic humoral compartments, respectively. Breast milk offers a unique opportunity to isolate sufficient mucosal IgG and IgA for both antigen binding and functional analysis. Thus, we isolated IgA and IgG separately from each of these compartments to allow isotype-specific interrogation. We first analyzed antigen specificity to a large multiclade panel of Env glycoproteins (gp120, gp140, and gp41), conformational scaffolds (gp70 V1/V2 and gp70 V3), and linear peptides (V2, V3, C5, and gp41 PID). Next, we analyzed function, focusing on virus neutralization, inhibition of epithelial cell binding, and phagocytosis of both HIV-1 Env-coated beads and infectious HIV-1 virions. We observed little correlation between the specificity and function of IgA and IgG isotypes (regardless of milk or plasma); such compartmentalization suggests that IgA and IgG have important differences in their mechanisms of regulation and induction. Such differences have also been described in other studies; for instance, in acute HIV-1 infection, gp41-specific IgA increases and then declines, whereas gp41-specific IgG continues to increase in magnitude across the same time points (34). Differences between isotypes have also been reported in the context of highly exposed seronegative persons, where IgA but not IgG is induced (40–45, 95). Three scenarios may explain the antigen specificity differences between isotype compartments. First, these differences may arise stochastically. Class switch fates are strongly correlated among closely related B cells (46); if cells with similar VDJ sequences and therefore antigen specificities switched in a coordinated manner to a particular isotype (either IgA or IgG), this would result in antigen specificity differences between isotypes. Second, these differences may arise from interactions between HIV-1 and the B cell class-switching process. Such interactions might involve direct action on the class-switching pathway, an example being the Nef-induced suppression of class switching leading to selective bias against IgA and IgG2 (47). Alternatively, they may involve action on the promoters of the constant region genes; it is known that the different isotype-defining constant region genes are controlled by different promoters, which respond to different cytokine and microbial product stimuli. For instance, the IgA constant region gene, Cα, has a promoter responsive to transforming growth factor β (TGF-β), whereas the IgG1 constant region gene, Cγ1, has a Stat6 promoter responsive to interleukin-4 (IL-4) (48). Third, these differences may arise from distinctions in B cell homing to mucosal surfaces (49), which may then lead to effects on antibody repertoire and/or class switching due to differential tissue microenvironment signals.

Mucosal compartments are known to differ in the IgA/IgG ratio from plasma. Since IgA and IgG antigen specificities differ, the IgA/IgG ratio likely also contributes to antigen specificity and dominance differences between mucosal and systemic compartments. In particular, in settings where IgA is dominant in mucosa while IgG is dominant in the systemic compartment, differential regulation of antigen specificities between antibody isotypes may be a mechanism of altering mucosal responses relative to systemic responses. Such settings include HIV-1 vaccination (50) or highly exposed persistently seronegative persons (44), as well as immune responses against other pathogens (51).

Examining the relationship between milk and plasma, we confirmed that milk and plasma IgGs were very highly correlated across a wide range of antigens (33), as correlations were found between milk and plasma across all specificities tested, including IgG V1/V2-, V2 linear-, V3-, C5 linear-, gp120-, gp140-, and gp41-specific responses. Yet this was not the case for milk and plasma IgAs, which were moderately correlated for gp140 and gp41 but uncorrelated for gp120. The tight relationship between mucosal and serum IgGs, but not between mucosal and serum IgAs, has also been found in the context of other mucosal pathogens, including Helicobacter pylori infection (52) and measles vaccination (dependent on route of inoculation) (53), and even in mucosal autoimmune diseases such as pemphigus vulgaris (54) but is not universal. In the context of mucosal vaccination, elicited mucosal IgA responses can be independent of, or moderately correlated with, systemic immunity (53, 55). One theory may be that systemic inoculation results in a systemic IgG response that transudates in a concentration-dependent manner into the breast milk compartment, resulting in a correlated mucosal IgG response. Continuity between mucosal and systemic IgG in general may explain why various mucosal pathogens have good correlates of risk with systemic antibodies in the context of systemic vaccination.

There are several implications of the finding of an antigen specificity difference between breast milk and systemic IgA responses. First, this suggests an additional layer of control for mucosal IgA. The mechanism remains unknown and in the case of milk may involve either selective transfer of IgA into the milk compartment or local production of IgA by plasma cells adjacent to the ductal epithelium (56) although it is notable that most memory B cells present in milk are of IgG isotype (57). Second, Env gp120 and gp140 plasma IgAs correlated with increased HIV-1 risk (i.e., decreased vaccine efficacy) in the RV144 trial (29), whereas mucosal IgA has been associated with a beneficial decreased risk of transmission in the BAN study (22) and also with protection from challenge in a nonhuman primate passive immunization model (16, 28). Our findings support the idea that these differences in protective effects may be due to differences in antigen specificities, in addition to the differences in abundances of each form of IgA in each compartment (monomeric versus dimeric/secretory) and differences in local interactions with effector cells. Notably, even in RV144, not all specificities of plasma IgA may have contributed to the finding of increased HIV-1 risk (i.e., reduced vaccine efficacy) (30). For instance, plasma IgA directed to the gp120 C1-C2 region blocked the ADCC activity of corresponding IgG (30), whereas it is unknown if other plasma IgA specificities (CD4 binding site, V3) were present that can block ADCC activity (58).

Using breast milk as a mucosal antibody source, we generated adequate amounts of mucosal antibody to determine neutralizing and nonneutralizing antibody effector functions, which is typically not possible with the small amount of other mucosal antibody sources. Despite the large quantity of milk HIV-specific IgA isolated, as well as detectable HIV-specific binding titers to multiple HIV-1 antigens, milk IgA showed little neutralization or phagocytosis function although epithelial cell binding inhibition activity was strong for one participant. With the high concentration of IgA isolated from milk (up to 5.87 mg/ml), we confirmed that the rarity of these functions in mucosal IgA is not simply due to a lack of antibody quantity.

Neutralization, epithelial cell binding inhibition, and phagocytosis functions were chosen for study in this paper because it has been shown in other contexts that IgA can mediate these functions (39, 59–61). For instance, IgA-mediated neutralization and epithelial cell binding inhibition have been seen for the mucosal secretions of highly exposed persistently seronegative women (40–45). For phagocytosis, mIgA1- and mIgA2-backbone versions of the CD4-binding site antibody CH31 mediated phagocytosis of ConS gp140-conjugated beads and were not inferior to the IgG1 backbone (39). However, we rarely found these functions in both milk and plasma HIV-specific IgA, whereas neutralization and phagocytosis activity were found for both milk and plasma HIV-specific IgG. This was not due to absence of the FcαRI receptor since expression of FcαRI did not enhance neutralization (Fig. 5) and since FcαRI is present on the human primary monocytes used for phagocytosis (39). Also, lack of phagocytosis activity in milk IgA cannot be attributed entirely to the inability of sIgA (which comprises the majority of milk IgA) to mediate phagocytosis (9–11) since a similar lack of phagocytosis activity was observed in plasma IgA, where sIgA is uncommon. These results support other studies of IgA from HIV-1-infected persons, which have also found poorer IgA neutralization function than IgG (27, 62).

Given the differences in IgA and IgG antigen specificity profiles described above, one potential explanation for this phenomenon is that the dominant antigen specificities in infection-induced IgA are nonfunctional for the above-listed functions, whereas IgG has dominant specificities that are functional. For instance, in our cohort, antigen specificity may have contributed to a difference in functions between isotypes; we observed a higher gp140/gp41 ratio in IgG than in IgA, and binding to gp140 but not gp41 was correlated with function. Interestingly, an Ad26/Env vaccination regimen in nonhuman primates was capable of eliciting IgA and IgG responses which correlated in magnitude and in linear antigen specificities, indicating that at least some modes of vaccination may circumvent the elicitation of different IgA and IgG antigen specificities that we have described in natural infection (63). Furthermore, the targeting of these antigen specificities may increase IgA function since the study found that neutralization function was correlated with both IgA and IgG titers. However, it is notable that the antigen specificities relevant for function may differ across contexts: gp41 antibodies have been implicated in neutralization function in highly exposed persistently seronegative women, in contrast to the gp120 or gp140 antibody-associated functions here (44, 64).

Differences in antigen specificities may not account for all functional difference, however, since even with the same specificity, it is still possible for IgA and IgG to differ in activities due to Fab-Fc interactions (65–68). For instance, when isotype-switched IgA and IgG versions of the broadly neutralizing CD4 binding site (CD4bs) antibodies b12 (61) and CH31 (69, 70) were compared, IgA had decreased neutralization activity relative to that of IgG. In contrast, when isotype-switched IgA and IgG versions of the CD4-induced (CD4i) antibody F425A1g8 were compared for neutralization, IgA was superior to IgG for neutralization activity (59). Further investigation into the reasons for functional impairment of IgA from HIV-infected individuals as seen in these assays, including effects of differential antigen specificity profiles and Fab-Fc interaction, is necessary.

Nevertheless, given the rarity of neutralization and phagocytosis functions in milk IgA, especially relative to activities of IgG, it is unlikely that these functions mediate the specific milk IgA (and not IgG) protection that has been found in some studies (22). Further investigations into other functions of IgA and sIgA, including more sensitive assays for epithelial cell binding inhibition and also other known functions of immune exclusion (71, 72) and intracellular neutralization (60, 73), are required to understand the role of IgA in mucosal immune defense. Such studies may also need to take into account immune complex size and composition, which may influence FcR binding and function (74, 75). Furthermore, interactions with local immune factors in the infant gut, especially mucins, may be critical to the *in vivo* function of breast milk IgA. In addition, in the context of HIV-1, determining the antigen specificities that correlate with such functions will inform vaccine design and passive immunotherapy approaches if these functions are crucial for protection.

In summary, we confirmed that anti-HIV-1 antibody antigen specificities and functions in breast milk and plasma did not correlate across IgG and IgA isotypes and that only IgG, but not IgA, correlated well across milk and plasma. Moreover, antibody functions were largely associated with IgG anti-gp120 activity and correlated across milk and plasma compartments. Finally, despite isolating high concentrations of purified systemic and mucosal IgA, we were unable to measure a consistent antiviral function. Additional functional assays are required to determine the mechanism of IgA anti-HIV-1 function. Understanding the ontogeny of antiviral mucosal and systemic IgA and IgG repertoires will inform vaccination approaches targeted toward mucosal pathogens.

## MATERIALS AND METHODS

### Study cohort.

Maternal breast milk (*n* = 20) and plasma (*n* = 20) samples were obtained from a cohort of pregnant women that tested HIV-1 positive during pregnancy as part of the CHAVI 009 protocol that recruited from two rural health clinics outside Blantyre, Malawi, between 2007 and 2009, at delivery, as previously described (33). Women were enrolled if breastfeeding was initiated. Approximately half of women were started on antiretrovirals (ARVs) in pregnancy according to local policies at the time of initiating ARVs if the peripheral CD4^+^ T cell count was below 350. A single dose of nevirapine was also administered to all mothers and infants at delivery. Twenty patients were chosen based on a high magnitude of Env-specific IgA responses measured in IgG-depleted breast milk from a total of 78 subjects. Women with an Env binding IgA magnitude in a binding antibody multiplex assay (BAMA) above a mean fluorescence intensity (MFI) of 5,000 against gp120 or gp41 were selected for IgA purification (Table 1), and of these, 16 participants yielded sufficient IgA for binding and phagocytosis analyses. Of these 16, 1 participant had insufficient sample for plasma IgG, and 1 participant had insufficient sample for milk IgG for binding analyses. This study was approved by the College of Medicine Research and Ethics Committee in Malawi and institutional review boards at Duke University Medical School where samples were received and processed for immune analysis. Breast milk and plasma viral load, peripheral CD4^+^ T cell count, and preliminary milk Env-specific IgA binding responses of the selected cohort are displayed in Table 1.

**TABLE 1:**
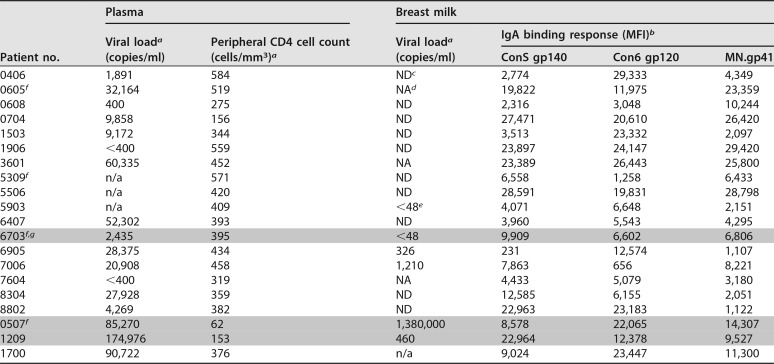
Plasma and milk viral loads, peripheral CD4^+^ T cell counts, transmission status, and HIV Env-specific IgA responses in unfractionated plasma and breast milk of the CHAVI 009 cohort

*^a^*Plasma viral load and peripheral CD4 T cell counts were determined upon enrollment in the third trimester.

*^b^*MFI, mean fluorescence intensity.

*^c^*ND, not detectable at a dilution of 1:5 (breast milk).

*^d^*NA, not available.

*^e^*Viral load was detectable but too low to quantitate (<48 copies/ml) at a dilution of 1:5 (breast milk).

*^f^*Sufficient yield in IgA and IgG milk and plasma only for neutralization analysis.

*^g^*Gray rows indicate transmitting mothers (6703 and 0507, in utero; 1209, postnatal).

### Breast milk delipidization and IgG purification.

A total of 10 ml of breast milk per patient was delipidized before IgA purification by centrifugation at 25,000 × *g* for 30 min at 4°C and then filtered with Spin-X filter columns (Fisher) as described previously (33). Delipidized milk was then concentrated using Amicon Ultra-4 filters (Millipore) to a final volume between 400 and 600 μl. Delipidized, filtered, and concentrated breast milk and 250 μl of corresponding plasma were IgG depleted using protein G resin prepacked into 96-well depletion plates (GE Healthcare), as previously described (76). Briefly, plasma samples were centrifuged at 10,000 × *g* for 10 min and then diluted 2-fold with Tris-buffered saline (TBS), pH 7.5. A total of 200 μl of the diluted sample was added in each well. Plates were incubated at room temperature for 1 h with shaking. Unbound fractions were removed by centrifugation at 700 × *g* for 3 min. Wells were then washed three times with 400 μl of TBS to remove loosely bound material. Bound IgG was eluted with 200 μl of 2.5% glacial acetic acid, pH 2.53, and then neutralized with 120 μl of 1 M Tris-HCl, pH 9.0. IgG fractions were collected, concentrated using Amicon Ultra-4 filters, and used as comparisons in select functional assays.

### IgA purification.

IgA was purified from IgG-depleted breast milk and plasma using peptide M resin (Invivogen) as previously described (76). Briefly, 200 μl of peptide M gel slurry was added to spin columns (Thermo Scientific). Following two washes with 500 μl of phosphate-buffered saline (PBS), IgG-depleted milk and plasma were added to the columns and incubated for 45 min at room temperature. Columns were spun and washed, and flowthrough was collected. Bound IgA was eluted with elution buffer (Thermo Scientific) and then neutralized with 120 μl of 1 M Tris-HCl, pH 9.0 (Thermo Scientific). Columns were washed and incubated overnight with PBS at 4°C, and eluted samples were run through the columns a second time. Eluted IgA fractions from both processes were combined, buffer exchanged, and then concentrated with Amicon Ultra-4 filters (Millipore). IgA concentration was determined via spectrophotometry at *A*_280_ using a NanoDrop 1000 spectrophotometer (Thermo Fisher Scientific). To confirm IgG depletion, samples were tested by human IgG enzyme-linked immunosorbent assay (ELISA) according to the manufacturer’s instructions (Immunology Consultants, Inc.). IgA preparations with IgG concentrations below 0.07 mg/ml were considered successfully depleted. The cutoff of 0.07 mg/ml was selected as the corresponding concentration of 3× the blank optical density (OD) of the total IgG ELISA.

### Virus production.

The original protocol for recombinant HIV-1 virus production has been previously described (77). Briefly, HIV-1 infectious molecular clone (IMC) variants were prepared by transfecting exponentially dividing 293T/17 cells with 4 μg of Rev/Env expression plasmid, using FuGENE 6 reagent (Promega) in growth medium, as described by the manufacturer. Virus stocks were titrated by performing serial 5-fold dilutions in quadruplicate in growth medium in 96-well culture plates (11 dilution steps total), and the 50% tissue culture infective dose (TCID_50_) was calculated per the method of Reed and Muench (78).The infectious viruses (subtype A/E, HIV-1_92Th023_-Tomato, subtype B HIV-1_BaL_-Tomato, and subtype C HIV-1_1086C_-Tomato) utilized in the phagocytosis assay were internally labeled via random incorporation of Vpr-Tomato (79) with no expected fluorescent molecules on the virus surface.

### Neutralization assays.

The ability of purified IgA and IgG milk and plasma fractions to neutralize the clade C tier 1 neutralization-sensitive HIV-1 IMCs MW965 and S0032 was measured in TZM-bl cells, as previously described (14, 80). To account for nonspecific activation of each sample, neutralization was also measured against murine leukemia virus SVA.MLV (23). Neutralization was measured after 48 h as a reduction in luciferase reporter gene expression (expressed in relative luminescence units [RLU]) compared with the level in the virus-only control. The 50% inhibitory concentration (IC_50_) titer was calculated as the milk or plasma IgA or IgG concentration that caused a 50% reduction in RLU compared to the level of virus control wells after subtraction of cell control RLU. Wells which exhibited visible toxicity, visualized as dead cells which had formed punctate bodies or cell aggregation, were excluded.

### TZM-bl cells transduced with FcαRI.

To allow the expression of FcαRI on TZM-bl cells, the cDNAs for the human FcαRI and the γ chain of the FcεR were transduced into TZM-bl cells using lentiviral constructs. The cDNAs for human FcRs were amplified by PCR using primers complementary to regions flanking the receptor open reading frame. PCR products were cloned in the lentiviral vector pLenti6/V5 under the control of a cytomegalovirus (CMV) promoter (Invitrogen). Recombinant retroviral vectors were packed into virions in 293FT cells and used to transduce TZM-bl cells. Transduced cells were selected in blasticidin-containing medium and live-sorted by flow cytometry using a fluorescein isothiocyanate (FITC)-conjugated anti-human CD89 monoclonal antibody (MAb). To detect surface expression of the FcαRI and FcRγε chains on this new TZM-bl cell line, TZM-bl cells expressing FcαR1 were removed from flasks using a nonenzymatic solution (Sigma) and washed twice with PBS. Approximately 2 × 10^6^ cells were stained with a FITC-conjugated mouse monoclonal antibody (MIP8a) to the human CD89 (Abcam, Inc., Cambridge, MA). Cells and antibody were incubated at 4°C for 30 min, washed twice with PBS, and fixed with PBS containing 1% formaldehyde. The levels of CD89 were increased significantly with the coexpression of the human FcεRγ chain. Similarly, phycoerythrin (PE)-conjugated monoclonal antibodies (BD Biosciences/BD Pharmingen, San Diego, CA) against the human CD4, CCR5, and CXCR4 receptors as well as QuantiBrite beads (Becton, Dickinson, San Jose, CA) were used to determine surface expression on the FcαR1 cell line. The cell line was fully susceptible to HIV infection as the levels of CD4, CCR5, and CXCR4 remained comparable to those of the parental cell line. To test IgA binding to TZM-bl/FcαRI-expressing cells, human IgA1(κ), IgA2(κ) (Athens Research and Technology, Athens GA), purified human secretory IgA (MP Biomedicals, LLC, Solon, Ohio), and FITC-conjugated goat anti-human IgA (Sigma) were used. Cells were washed in PBS containing 0.1% bovine serum albumin (BSA) and fixed in PBS containing 1% formaldehyde before analysis.

### BAMA of purified IgA and IgG fractions from milk and plasma.

The HIV-1 binding antibody multiplex assay (BAMA) was performed as previously described (20, 26, 81–84). Briefly, HIV-1 antigens were conjugated to polystyrene beads (Bio-Rad), and binding of Ig to the bead-conjugated HIV-1 proteins was measured in matched milk and plasma IgA and IgG preparations from 16 HIV^+^ women. Eleven seronegative milk and eight seronegative plasma samples were also tested. IgA was detected by goat anti-human IgA-PE (Jackson Immunoresearch), sIgA was detected by mouse monoclonal anti-human secretory component (clone GA-1; Sigma), and IgG was detected by mouse anti-human IgG-PE (Southern Biotech). Milk and plasma IgAs were diluted 1:10 in tests against Env proteins and 1:100 in tests against linear peptides. Milk IgG was diluted 1:10, 1:250, or 1:500 depending on antigen reactivity, and plasma IgG was diluted 1:50 or 1:5,000 depending on antigen reactivity. After incubation of beads and sample, bound IgA or IgG was detected with the appropriate secondary antibody; then the beads were washed and acquired on a Bio-Plex 200 instrument (Bio-Rad). HIV-1-specific binding antibody responses against each antigen were measured as mean fluorescence intensity (MFI). To control for nonspecific sample binding, the MFI of sample binding to unconjugated beads was subtracted from the MFI of each antigen. For antigens utilizing a gp70 scaffold, the MFI of sample binding to an empty scaffold (MulVgp70_His6) was subtracted from the MFI of each antigen. Positive controls included pooled purified HIV^+^ Ig (HIVIG; NIH), CH58 IgG (anti-V1/V2 MAb), and 7B2 sIgA (anti-gp41 MAb). Normal human serum was utilized as a negative binding control, and 7B2 mIgA and dIgA were utilized as negative sIgA detection controls. The data were normalized to total IgA or IgG concentration as specific activity (MFI ×dilution/amount of purified Ig [in micrograms per milliliter]). Samples with MFI values above 100 were considered positive. Overall gp41, gp120, gp140, and V1/V2 binding scores were determined by taking the mean of panels of 6, 8, and 14 antigens with nonredundant antigenicity, respectively. Specifically, the gp41 binding score represented the average of binding to 2 gp41 antigens (gp41 clade C and gp41 clade B MN), the gp120 binding score represented the average of binding to 6 gp120 antigens (BORI_D11gp120, CNE20_D11gp120, TT31P.2792_D11gp120, A244 D11gp120, 254008_D11gp120, and B.6240_D11gp120), the gp140 binding score represented the average of binding to 8 gp140 antigens (RHPA4259_C7.gp140C, 1086 C gp140C, BF1266_gp140C, 9004S.gp140C, AE.01.con_env03 gp140CF, SC42261_gp140, C.CH505TF_gp140, and WITO4160.gp140C), and the V1/V2 binding score represented the average of binding to 10 gp70 V1/V2 antigens (gp70-CM244.ec1 V1/V2, gp70-BF1266_431a_V1/V2, gp70-BJOX002000.03.2 V1/V2, gp70-7060101641 V1/V2, gp70-96ZM651.02 V1/V2, gp70-001428.2.42 V1/V2, gp70_B.CaseA_V1_V2, gp70-191084_B7 V1/V2, gp70-700010058 V1/V2, gp70-C2101.c01_V1/V2, gp70-CAP210.2.00.E8 V1/V2, gp70-TT31P.2F10.2792 V1/V2, gp70-TV1.21 V1/V2, and gp70-Ce1086_B2 V1/V2) (85). Positivity to an antigen panel was defined by a readout of >100 MFI (background-subtracted) to at least half of the antigens tested. Binding responses to a clade B V3 gp70 scaffold, as well as to biotinylated linear peptides was also examined: gp41 PID (RVLAVERYLRDQQLLGIWGCSGKLICTTAVPWNASWSNKSLNKI) (34), V2 linear (TSIRDKVQKEYALFYKLDVVP), V3 linear (NNTRKSIRIGPGQTFYATGDIIGDIRQAHC), and C5.2 linear (SELYKYKVVKIEPLGVAPTKAKRRVVQREKRAV). For statistical analysis, background-subtracted MFI values below 0 were truncated to 0, and values above the linear range (23,000) were truncated to 23,000. If more than half the antigens within an antigen panel had MFI values below 100 or missing values, the antigen panel score for that participant was treated as a missing value.

### Inhibition of epithelial cell binding.

To determine the ability of milk and plasma purified IgAs and IgGs to impede infectious HIV-1 binding to colonic epithelial cells, a modified previously reported protocol was used (17, 61, 86). Colonic HT-29 cells (ATCC) were grown to confluence on a 96-well flat-bottom plate in modified McCoy’s 5A medium (Thermo Fisher Scientific) supplemented with 10% fetal bovine serum (FBS) and antibiotics. The HT-29 cells were washed once with serum-free medium and treated with 100 μl of 50 μg/ml mitomycin C for 1 h to prevent further division, followed by two washes. Then, 3.6 × 10^6^ TCID_50_ of HIV-1 Ce1086_B2.LucR.T2A.ecto/293T IMC was incubated with 5 μg/ml of purified milk or plasma IgA or IgG for 1 h at 37°C and then added in quadruplicate to the colonic epithelial cell monolayer. Plates were incubated at 37°C for 4 h. Monolayers were washed twice with PBS to remove free virus, and 1 × 10^4^ TZM-bl reporter cells were added to each well of the virus-bound monolayer. After 48 h, luciferase reagent (Bright-Glo; Promega) was added to the well, and the RLU was measured. Percent inhibition was calculated by dividing the number of RLU of each well by the median number of RLU of epithelial-bound virus that was not preincubated with Ig. The anti-influenza virus hemagglutinin (HA) MAb CH65 IgA and normal human colostrum IgA were used as negative controls, whereas the broadly neutralizing anti-HIV-1 CD4 binding site MAbs VRC01 IgA and IgG (87) were used as positive controls.

### Antibody-mediated phagocytosis assay.

The potency of milk and plasma purified IgAs and IgGs to mediate phagocytosis was tested using a previously published protocol (39, 88). Phagocytosis of 9 × 10^5^ HIV-1 Env (ConS gp140 or 1086.C gp140)-coated 1-μm FluoSpheres NeutrAvidin-labeled beads (ThermoFisher) or 10 μl of live infectious fluorescent HIV-1_92Th023_-Tomato (containing 2 to 20 ng of p24) was measured. Immune complexes were generated by incubating antibody-coated bead or virus with 0.5 μg of purified milk/plasma IgA or IgG antibodies (final 25-μg/ml concentration) at 37°C for 2 h. Frozen elutriated human primary monocytes were thawed and rested overnight at 37°C in RPMI medium with 10% fetal bovine serum (FBS) and 1% penicillin-streptomycin. One hour prior, CD4 was blocked as previously described to reduce background virus internalization levels (39). A total of 4 × 10^4^ to 5 × 10^4^ human primary monocytes were added to the immune complexes with a final concentration of 1.5 × 10^6^ cells/ml and then spinoculated for 1 h at 4°C and incubated for 1 h at 37°C to allow phagocytosis to occur. After incubation, supernatant was removed, and cells were washed with trypsin for 10 min at 37°C and fixed with 2% paraformaldehyde. For experiments without spinoculation, the spinoculation step was omitted and samples were transferred directly to the 37°C incubation for 1 h. The positivity cutoff was calculated for each antigen (ConS gp140, 2.51; 1086.C gp140 beads, 3.33; HIV-1_92Th023_-Tomato virions, 1.50) based on the mean +3 standard deviations of values of all negative-control antibodies, including 4 negative monoclonal antibody controls and 16 polyclonal antibodies derived from seronegative plasma and milk IgG and IgA. The phagocytosis score was calculated as previously described (39) and was equal to the percentage of cells with greater fluorescence than the 95th percentile of fluorescence from a no-antibody control, multiplied by their MFI and divided by the corresponding product for the no-antibody control.

**FIG 11:**
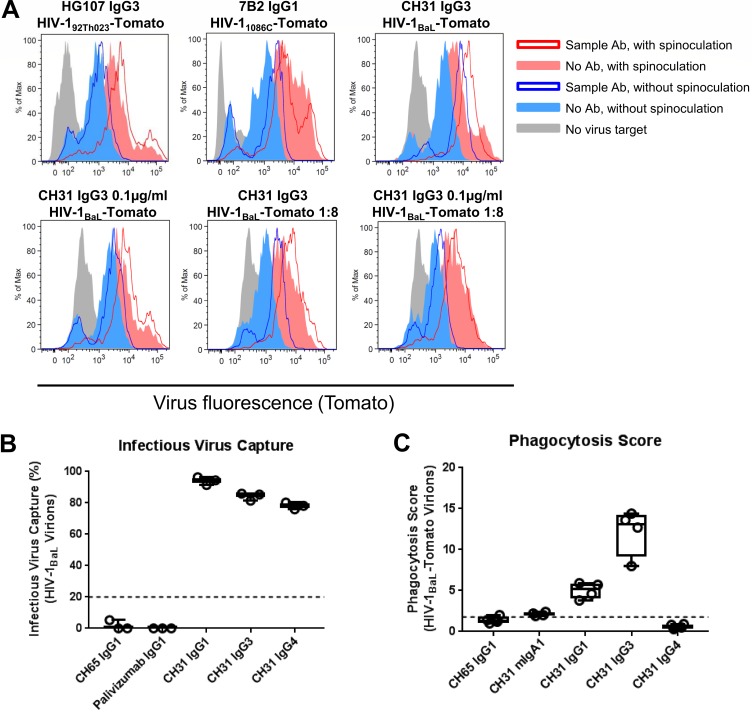
Virion phagocytosis is not dependent on spinoculation and requires specific Fc-FcR interaction. (A) To assess whether virion phagocytosis was dependent on the spinoculation procedure in the experimental setup, parallel conditions were set up with and without the spinoculation step (see Materials and Methods), as indicated in the legend on the figure. Spinoculation was performed at 1,200 × *g* for 1 h at 4°C. Antibodies were added at 12.5 μg/ml unless otherwise specified. Three pairs of antibody and virus were chosen based on their known properties for virion phagocytosis in the presence of spinoculation (top row). All three pairs retained virion phagocytosis signal even when spinoculation was omitted though overall specific and nonspecific virion phagocytosis was decreased relative to values for the experiments with spinoculation. This remained true despite decreasing the concentration of antibody (bottom left), virus (bottom-middle), or both antibody and virus (bottom right). (B and C**)** To further assess whether virion phagocytosis was dependent on Fc-FcR interactions, the CD4 binding site broadly neutralizing antibody CH31 was recombinantly expressed in human mIgA1, IgG1, IgG3, and IgG4 backbones and examined for virus capture of HIV-1BaL virions or phagocytosis of HIV-1BaL-Tomato virions. For virus capture, the median and range of three replicates from a single experiment are reported, and for phagocytosis, the median and range of data from four different human primary monocyte donors representing two independent experiments are reported. A positivity cutoff of 20% for infectious virus capture and 1.77 for virion phagocytosis was determined based on the mean +3 standard deviations of the values for historical negative controls. CH31 mIgA1, IgG1, and IgG3 mediated virion phagocytosis, as reported, while CH31 IgG4 did not, despite its ability to capture virions, showing that virion phagocytosis is dependent on Fc-FcR interactions.

To test that virion phagocytosis was not dependent on spinoculation (89), we examined phagocytosis of three different viruses (i.e., subtype A/E, HIV-1_92Th023_-Tomato, subtype B HIV-1_BaL_-Tomato, and subtype C HIV-1_1086C_-Tomato) with either a V1/V2 IgG3 MAb (HG107), gp41 IgG1 MAb, or a CD4bs IgG3 MAb (CH31) at multiple antibody and virus concentrations. For all virus-antibody combinations, positive virion phagocytosis was seen both with and without spinoculation, with lower levels of specific and nonspecific virion phagocytosis in the absence of spinoculation (Fig. 11A). As an additional negative control, we expressed the CD4 binding site broadly neutralizing antibody CH31 as an IgG4 antibody. CH31 IgG4 was able to capture virions (Fig. 11B), as measured by a protein G-based virus capture assay (84). However, consistent with the poor/lack of IgG4 binding to cellular FcR (90, 91), the presence of CH31 IgG4 in the immune complex did not result in virion phagocytosis (Fig. 11C). These data support the finding that the observed virion phagocytosis is dependent on a specific antibody Fc-FcR interaction.

### Statistical methods.

The IgA-, sIgA-, and IgG-related specific activity (SA) magnitudes were compared by using a nonparametric method, the Wilcoxon signed rank test. The correlations of paired SAs, functional responses, and their 95% confidence intervals were calculated using Kendall’s tau method, and Steiger’s *Z* test was applied for comparing correlations. In order to control type I error of multiple-comparison tests, all *P* values were adjusted with the false discovery rate (FDR)-controlled Benjamini-Hochberg method (92). Whenever there were missing data points for either partner of a pairwise comparison, the other partner was excluded from that analysis as well. All analyses were performed in R, version 3.4.2 (93), using the stats and cocor packages (94).
